# Therapeutic efficacy of the bromodomain inhibitor OTX015/MK-8628 in ALK-positive anaplastic large cell lymphoma: an alternative modality to overcome resistant phenotypes

**DOI:** 10.18632/oncotarget.12876

**Published:** 2016-10-25

**Authors:** Michela Boi, Maria Todaro, Valentina Vurchio, Shao Ning Yang, John Moon, Ivo Kwee, Andrea Rinaldi, Heng Pan, Ramona Crescenzo, Mangeng Cheng, Leandro Cerchietti, Olivier Elemento, Maria E. Riveiro, Esteban Cvitkovic, Francesco Bertoni, Giorgio Inghirami, The AIRC 5xMille Consortium ‘Genetics-Driven Targeted Management of Lymphoid Malignancies’

**Affiliations:** ^1^ Department of Molecular Biotechnology and Health Science and Center for Experimental Research and Medical Studies (CeRMS), University of Torino, Torino, Italy; ^2^ Department of Pathology and Laboratory Medicine, Weill Cornell Medical College, New York, NY, USA; ^3^ Joan and Sanford I. Weill Department of Medicine, Weill Cornell Medical College, New York, NY, USA; ^4^ Lymphoma and Genomics Research Program, IOR Institute of Oncology Research, Bellinzona, Switzerland; ^5^ Dalle Molle Institute for Artificial Intelligence (IDSIA), Manno, Switzerland; ^6^ Swiss Institute of Bioinformatics (SIB), Lausanne, Switzerland; ^7^ Institute for Computational Biomedicine, Department of Physiology and Biophysics, Weill Cornell Medical College, New York, NY, USA; ^8^ Institute for Precision Medicine, Weill Cornell Medical College, New York, NY, USA; ^9^ In Vitro Pharmacology, Merck Research Laboratory, Boston, MA, USA; ^10^ Oncology Therapeutic Development, Clichy, France; ^11^ Oncoethix SA (Now Oncoethix GmbH, A Wholly Owned Subsidiary of Merck Sharp & Dohme Corp.), Lucerne, Switzerland; ^12^ IOSI Oncology Institute of Southern Switzerland, Bellinzona, Switzerland; ^13^ Department of Pathology, and NYU Cancer Center, New York University School of Medicine, New York, NY, USA

**Keywords:** anaplastic large cell lymphoma, BRD inhibitor, OTX015/MK-8628, tyrosine kinase inhibitor, gene expression profiling

## Abstract

Anaplastic large cell lymphomas (ALCL) represent a peripheral T-cell lymphoma subgroup, stratified based on the presence or absence of anaplastic lymphoma kinase (ALK) chimeras. Although ALK-positive ALCLs have a more favorable outcome than ALK-negative ALCL, refractory and/or relapsed forms are common and novel treatments are needed. Here we investigated the therapeutic potential of a novel bromodomain inhibitor, OTX015/MK-8628 in ALK-positive ALCLs.

The effects of OTX015 on a panel of ALK+ ALCL cell lines was evaluated in terms of proliferation, cell cycle and downstream signaling, including gene expression profiling analyses. Synergy was tested with combination targeted therapies.

Bromodomain inhibition with OTX015 led primarily to ALCL cell cycle arrest in a dose-dependent manner, along with downregulation of MYC and its downstream regulated genes. MYC overexpression did not compensate this OTX015-mediated phenotype. Transcriptomic analysis of OTX015-treated ALCL cells identified a gene signature common to various hematologic malignancies treated with bromodomain inhibitors, notably large cell lymphoma. OTX015-modulated genes included transcription factors (*E2F2*, *NFKBIZ, FOS*, *JUNB, ID1, HOXA5 and HOXC6*), members of multiple signaling pathways (*ITK*, *PRKCH, and MKNK2*), and histones (clusters 1-3). Combination of OTX015 with the Bruton's tyrosine kinase (BTK) inhibitor ibrutinib led to cell cycle arrest then cell death, and combination with suboptimal doses of the ALK inhibitor CEP28122 caused cell cycle arrest. When OTX015 was associated with GANT61, a selective GLI1/2 inhibitor, C1156Y-resistant ALK ALCL growth was impaired.

These findings support OTX015 clinical trials in refractory ALCL in combination with inhibitors of interleukin-2-inducible kinase or SHH/GLI1.

## INTRODUCTION

Peripheral T-cell lymphomas (PTCL) are a heterogeneous group of lymphomas whose pathogenetic mechanisms of transformation remain largely undefined. Clinically, PTCL patients are often refractory to conventional therapies and survival is dismal. Anaplastic large cell lymphomas (ALCLs) represent a relatively common subset of PTCL, comprising approximately 10% to 15% of all pediatric and adolescent lymphomas and 2-5% of adult non-Hodgkin lymphomas. Many ALCL are driven by recurrent translocations involving the anaplastic lymphoma kinase (ALK) gene (ALK+). Conversely, ALK-negative (ALK-) ALCLs display alternative alterations including the t(6;7) translocation [1], activating JAK/STAT mutations [2], or express truncated forms of the ERBB4 gene [3]. Many ALCLs display single or concomitant loss of the *BLIMP1* and *TP53* genes in both ALK+ and ALK- tumors [4].

In ALK+ ALCL, ALK fusions lead to the activation of several signaling pathways (JAK-STAT, Ras, PI3k-ATK, etc.) and robust c-MYC expression [5, 6]. The sonic hedgehog (SHH)/GLI1 signaling pathway also plays a role in ALK+ ALCL. SHH signaling is enhanced by activation of the PI3K/AKT pathway, and high and stable levels of GLI1 are pathogenetic. Conversely, downregulation of SHH/GLI1 signaling is linked to poor cell viability and decreased clonogenicity [7].

ALCL patients are most commonly treated with CHOP-based treatments (cyclophosphamide, doxorubicin, vincristine, prednisone) and radiotherapy. In ALK+ ALCL, ALK inhibitors (ALKi) represent a potentially effective treatment strategy [5, 8], although drug resistance inevitably develops [9]. This latter scenario may be managed by second or third generation ALKi [10] or by drugs targeting alternative signaling pathways, such as PI3K/Akt1/mTOR, JAK/Stat3 and RAS/ERK.

Changes in the complex epigenetic code are frequently a critical element in the development of cancer and are required for maintaining neoplastic phenotypes [11]. This phenomenon provides a rationale for developing new compounds or for the clinical implementation of existing agents targeting chromatin-modifying enzymes, such as DNA methyltransferases and histone deacetylases (HDACs), which have shown some clinical efficacy. Such agents have been introduced into the hematologic armamentarium, particularly in the management of T-cell lymphomas [12].

Among post-translational modifications, histone lysine acetylation plays an important role in the orderly control of gene transcription. It biophysically facilitates chromatin opening and recruits an emerging class of co-activators, ‘readers’ that recognize ε-acetyl lysine through a specialized recognition motif, the bromodomain (BRD) (14). Readers assemble transcriptional complexes at enhancer or promoter sites that initiate and regulate gene transcription [11]. The family of bromodomain and extra terminal (BET) proteins, which include the BRD2, BRD3 and BRD4 proteins, belongs to a larger family of the reader proteins [13]. BET proteins couple histone acetylation to transcript elongation, in particular for growth and survival genes, such as c-MYC, rendering BET inhibitors a promising class of anticancer agents. Several BET protein small molecule inhibitors are under development, including JQ1, I-BET151, CPI-0610 and GSK525762 [14, 15]. Mechanistically, these compounds displace BET proteins from the chromatin and by consequence the associated transcript initiation and elongation factors [16, 17]. This selectively interferes with various gene expression programs, supporting the potential use of this class of compounds in a variety of arenas, with efficacy against many cancers in *in vitro* and *in vivo* models already demonstrated [16–22].

OTX015 (MK-8628), a novel oral BET inhibitor in early clinical development, has shown preclinical activity against a wide range of hematologic malignancies [22, 23] as well as both pediatric and adult solid tumors [24, 25]. Here we present *in vitro* data probing its mechanism of action in ALCL models and demonstrating its potential therapeutic efficacy in treating ALCL, alone and in combination with agents employed in the clinical ALK+ setting.

## RESULTS

### OTX015 has antiproliferative activity in in vitro ALK-positive ALCL models

**Figure 1:**
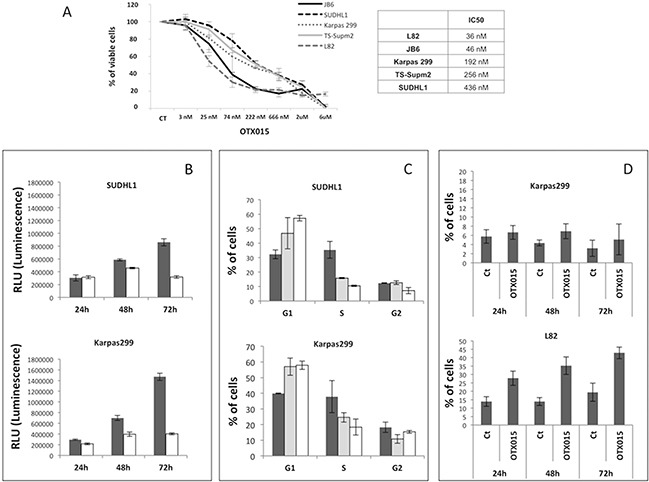
OTX015 displays antiproliferative activity in ALK-positive ALCL in vitro models **A.** The antiproliferative activity of OTX015 was determined with the MTT assay in a panel of ALK+ ALCL cell lines treated for 72 h with a range of OTX015 doses (3 nM to 6 μM) according to IC50 values. CT, DMSO-treated controls. **B.** Antiproliferative activity was evaluated using the ATPlite assay after 24, 48 and 72 h exposure to 500 nM OTX015 (solid color bar corresponds to vehicle [DMSO], open bar to OTX015). **C.** FACScan flow cytometry showed that 24 h OTX015 exposure (250 and 500 nM) led to G1 cell cycle arrest in all cell lines with the exception of L82 (solid color bar corresponds to vehicle [DMSO], dotted bar to 250 nM and open bar to 500 nM). **D.** Percent apoptosis after 24, 48 and 72-h exposure to 500 nM OTX015. Long exposure (72 h) was associated with an increased rate of apoptosis. Ct, DMSO-treated control.

Previous studies demonstrating that OTX015 has pre-clinical activity in B-cell lymphomas [22] were extended here to T-cell lymphomas, using a panel of five ALK+ ALCL cell lines (SUDHL1, TS-Supm2, L82, DEL, Karpass 299 and JB6). We first evaluated the antiproliferative activity of OTX015 in this panel and estimated their IC50 values demonstrating that the L82 and JB6 cell lines were the most sensitive. The median IC50 of the panel was 192 nM, with a range of 36 nM to 436 nM (Figure 1A). Although no significant changes in cell viability were observed at the earliest time point evaluated (24 h), after 48 h cell proliferation block was detected in all cell lines which was more pronounced after 72 h (Figure 1B). Of note, evaluation of the percentage of G1 cells showed that short exposure (24 h) was sufficient to induce cycle arrest in the absence of a detectable fraction of hypodiploid cells (Figure 1C and data not shown) in all cell lines other than L82 in which early cell death was seen (Figure 1D). Interestingly, prolonged exposure to OTX015 (120-144 h) led to a senescent-like phenotype (Supplementary Figure S1A), a finding confirmed by the presence of increased mRNA expression of *NANOG* and *OCT4*, two transcription factors involved in the self-renewal of embryonic stem cells (Supplementary Figure S1B).

### MYC and BRD expression is modulated after OTX015 treatment

**Figure 2:**
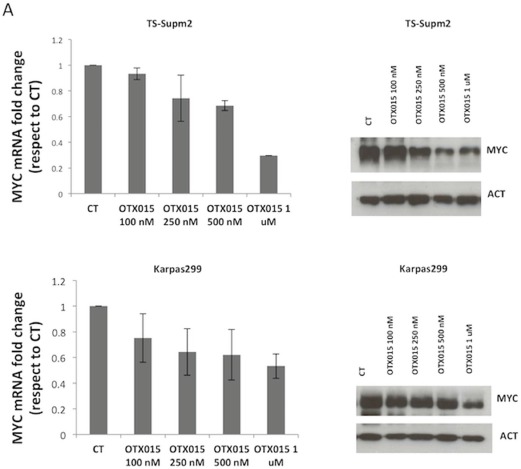

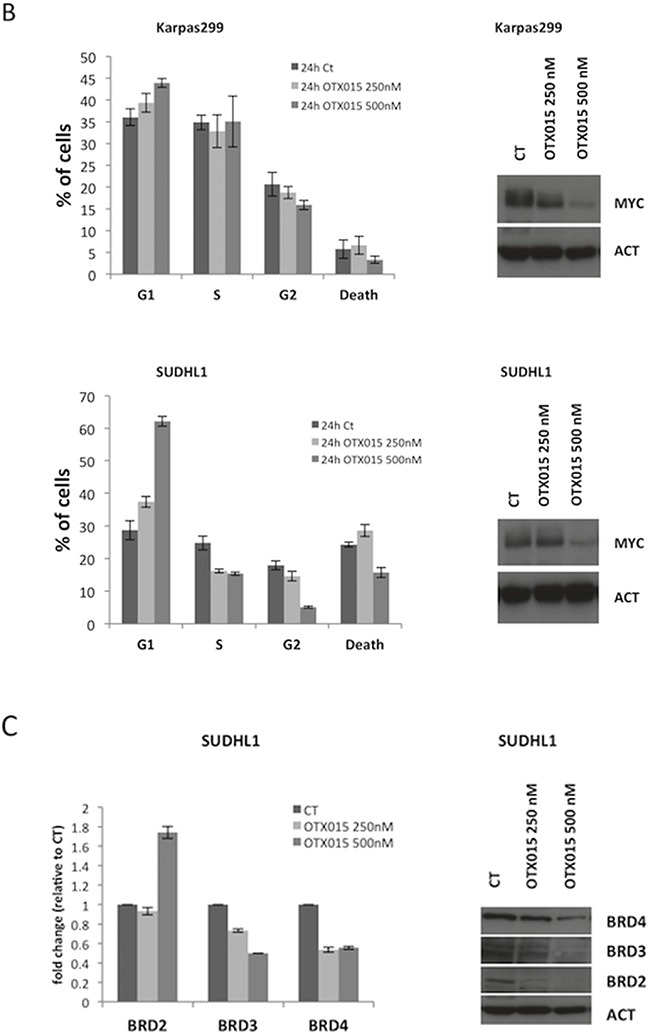
OTX015 modulates MYC and BRD expression **A.** MYC mRNA and protein levels were reproducibly down-regulated after 72-h OTX015 exposure (100 - 1000 nM) in ALK+ cell lines by qRT-PCR and Western blot respectively. CT, DMSO-treated controls. **B.** 24 h exposure to OTX015 (250, 500 nM) led to dose-dependent down-regulation of MYC protein levels and G1 cell cycle arrest. β-actin was used as a protein loading control. **C.** 24 h exposure to OTX015 resulted in down-regulation of BRD2, BRD3 and BRD4 RNA and protein levels by qRT-PCR and Western blot. β-actin was used as a protein loading control.

To gain an understanding of the mechanism of action of OTX015 in ALCL, we evaluated MYC levels following drug exposure. As reported for other BET inhibitors [16, 17, 22], OTX015 down-regulated MYC mRNA and protein levels in a dose-dependent manner after 72 h exposure (Figure 2A). This was associated with strong protein down-regulation occurring as early as 24 h after OTX015 exposure and cell cycle block (Figure 2B).

We then compared the effect on MYC levels of a single exposure to OTX015 (250 or 500 nM) over 72 h with repeated exposure every 24 h for 72 h. MYC down-regulation and changes to G1 cell cycle arrest were comparable after 72 h in both settings, suggesting that a maximum *in vitro* effect can be achieved with a single 72-h exposure (Supplementary Figure S2A). A series of washout experiments were also performed in which ALCL cells were treated with OTX015 for 12 h, then washed and cultured for up to 48 h. MYC mRNA expression was reduced after 12 h and remained so for up to 24 h after washout, returning to basal levels after 48 h (Supplementary Figure S2B).

Comparison of OTX015 and JQ1, an analog of OTX015 and a bona fide BRD inhibitor, whose mechanism of action and efficacy have been extensively validated *in vitro* [16, 18], demonstrated comparable MYC mRNA and protein down-regulation (Supplementary Figure S3A and S3B).

Effects of OTX015 on downstream MYC signaling were evaluated. The down-regulation of MYC mRNA following OTX015 exposure was associated with decreased CAD and NUC mRNA expression and concomitant up-regulation of ODC, all of which are known MYC targets [26], as anticipated from other studies [16, 22, 23] (Supplementary Figure S2C). We extended these analyses to evaluate the effect of OTX015 exposure on BRD expression, demonstrating a dose-dependent down-regulation of both mRNA and protein levels of BRD2, BRD3, and BRD4, with the exception of BRD2 mRNA (Figure 2C).

**Figure 3:**
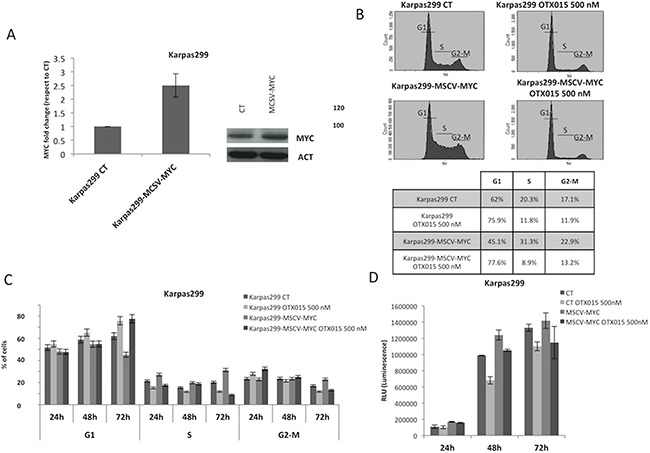
MYC overexpression does not counteract the OTX015-mediated phenotype **A.** Lentiviral transduction of Karpas299 ALK+ ALCL cells with the MSCV-MYC gene resulted in forced MYC mRNA and protein overexpression, as evaluated by qRT-PCR (left panel) and Western blot (right panel). **B-C.** Cell cycle analyses showed growth of wild type Karpas299 and Karpas299-MSCV-MYC cells was arrested after OTX015 exposure (500 nM for 72 h), as evaluated by FACScan. **D.** Antiproliferative activity of 500 nM OTX015 in wild type Karpas299 and Karpas299-MSCV-MYC cells was evaluated by ATPlite after 24, 48 and 72 h drug exposure.

To further understand the role of MYC in BRD-mediated cell growth regulation, we transduced Karpas299 cells with a lentiviral cassette coding for human MYC under an ectopic MSCV promoter (Figure 3A). When exposed to OTX015 (500 nM for 72 h), control (wild type Karpas299) and MSCV-MYC Karpas299 cells displayed similar cell cycle profiles (Figure 3B), although transfected cells overexpressing MYC expression took longer to accumulate in G1 arrest (Figure 3B), and displayed cell growth inhibition with OTX015 (Figure 3C–3D). mRNA levels of CAD and NUC paralleled MYC expression (data not shown). These data demonstrate that MYC overexpression delays but does not compensate the OTX015-mediated phenotype (i.e., growth inhibition and modified levels of downstream actors), suggesting that the biological changes imposed by OTX015 are linked to a multi-gene regulation scenario.

### OTX015 affects the ALK+ ALCL transcriptome

**Figure 4:**
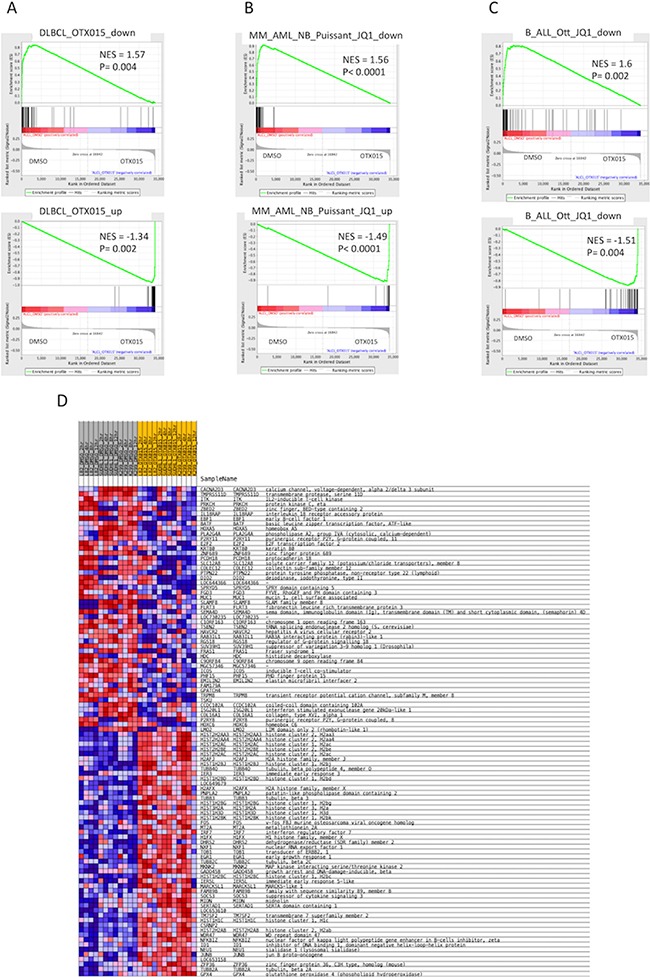
OTX015 affects the transcriptome of ALK+ ALCL cells, partially recapitulating gene expression changes observed in other preclinical cancer models exposed to OTX015 or JQ1 Gene expression profiling followed by gene set enrichment analysis was performed in three ALK+ ALCL cell lines (SUDHL1, Karpas299, L82) after exposure to DMSO or OTX015 (500 nM) for 2, 4, 8 or 12 h. Enrichment plots for gene sets obtained in **A.** DLBCL cells exposed to OTX015 [22], **B.** multiple myeloma, acute myeloid leukemias, neuroblastoma [20] treated with JQ1 and **C.** in B-cell acute lymphoblastic leukemia cells exposed to JQ1 [36]. NES = normalized enrichment score. **D.** The heatmap shows the top 50 up-regulated (upper part) and top 50 down-regulated (lower part) following treatment with OTX015. Samples labeled in grey are DMSO-treated control cells and samples labelled in yellow are OTX015-treated cells, for all three cell lines and at each time point. Red denotes high expression, blue denotes low expression.

To broaden our knowledge of the mechanism of action of OTX015, we performed a gene expression profiling (GEP) study on three ALCL cell lines (L82, SUDHL1, Karpas299) exposed to DMSO or OTX015 (500 nM) for 2, 4, 8 or 12 h, followed by a functional analysis using GSEA. We first looked at genome-wide changes induced by OTX015 and found that the changes seen in our ALCL cell lines displayed a high degree of overlap with the signatures previously reported in B-cell lymphomas treated with the same compound [22] (Figure 4A) or with the BET inhibitor JQ1 in other hematologic malignancy models (Figure 4B–4C) [20, 44]. Accordingly, up-regulated transcripts were mainly enriched for genes involved in chromosome/telomere maintenance, the cell cycle, the p53 pathway, or for genes up-regulated by HDAC inhibitors (Figure 4D). Genes involved in the TNFα via NFKB pathway and in PI3K or mTOR signaling were also enriched (Supplementary Tables S3–S5). In addition to MYC targets, down-regulated transcripts included E2F targets, genes involved in interferon alpha responses, the cell cycle, RNA metabolism, and genes down-regulated by HDAC inhibitors (Supplementary Tables S3–S5). To assess the capacity of OTX015 to interfere with the pathogenetic mechanisms driving ALK+ ALCL, we looked at its effect on the ALCL-derived ALK and STAT3 gene expression signatures [27, 28]. We found that only the latter profile was significantly enriched among the genes down-regulated by OTX015 (Supplementary Figures S4-S5).

We then searched for individual genes whose expression levels were affected by OTX015. Supplementary Table S5 and Figure 4D show the top-50 ranked down- and up-regulated genes, all but one of which were also differentially expressed applying a Limma t-test with an adjusted p-value < 0.05. Among the down-regulated genes were *ITK*, *E2F2*, *PRKCH* (protein kinase C), *IL18RAP*, *EBF1*, *ICOS*, *EMILIN2*, *HOXA5*, *HOXC6*, and *LMO2*. Conversely, genes encoding histones (clusters 1-3), *H2AFX*, *GADD45B*, *FOS*, *JUNB*, *MKNK2*, *ID1*, and *NFKBIZ* were up-regulated.

### OTX015 is synergistic with drugs acting via alternative pathways

Given the cytostatic nature of OTX015, we also wished to identify novel potentially effective combinations in the lymphoma setting. ALCL cells were exposed for 72 h to OTX015 (500 nM) alone or in combination with one of an array of 38 drugs targeting pathogenetic pathways or known to be effective in ALCL/PTCL (Supplementary Table S2), either concomitantly or initially with OTX015 (500 nM, up to 72 h) followed by a challenge with the additional compound. On the basis of the initial screening analysis (Supplementary Table S2; data not shown), we concomitantly treated the panel of ALCL cell lines with OTX015 (500 nM for 72 h) and a broad range of concentrations of eight compounds (31.25 nM to 10 μM for 72 h) (data not shown; Supplementary Table S6).

As reported in Supplementary Table S6 the combination of a second compound gave additive/synergistic effects with most of the eight drugs evaluated, with the combination of OTX015 and the drug giving reduced IC50 values in many settings. Overall, effective cell growth inhibition was reached by the combination of OTX015 and relatively low doses of several drugs.

**Figure 5:**
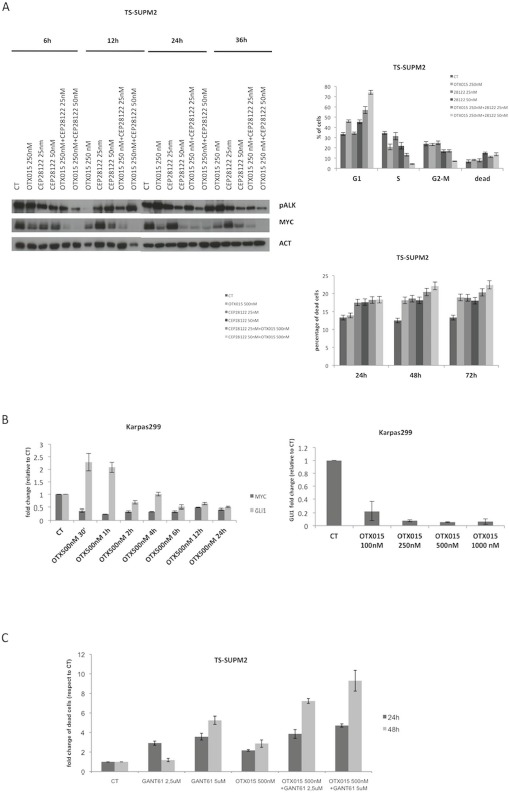

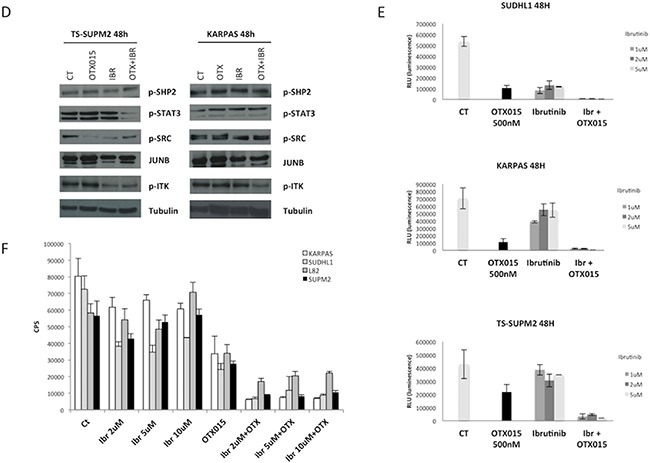
Cooperative effects of OTX015 with a panel of drugs in ALCL *in vitro* models **A.** The combination of OTX015-CEP28122 effectively down-regulated MYC protein levels by Western blot in TS-Supm2 cells (β-actin was used as a loading control), resulting in a pronounced G1 cell cycle arrest with limited effects on cell viability according to FACScan. The effect was maximal after 48 h. **B.** OTX015 exposure (500 nM) for 30 minutes to 24 h in Karpas299 cells resulted in robust down-regulation of GLI1 mRNA, a known target of GANT61, following an initial rapid upregulation according to qRT-PCR. Exposure of in Karpas299 cells to increasing OTX015 concentrations for 24 h showed that the mRNA down-regulation was dose-dependent. **C.** The effects on cell viability of the GLI inhibitor GANT61 and OTX015 were measured as single agents and in combination according to the qRT-PCR. Concomitant exposure of OTX015 (500 nM) and GANT61 (2.5 or 5 μM) increased the percentage of dead cells after 24 and 48 h exposure. **D.** Cells treated with combined ibrutinib (10 μM) and OTX015 (500 nM) had reduced levels of P-STAT3 and P-ITK protein compared to treatment with either single agent, as per Western blot. β-actin was used as a loading control. **E.** Metabolic readout of ALCL cell lines treated with OTX015 (500 nM) in combination with ibrutinib (1.2 or 5 μM) for 48 h, determined using an ATPlite assay. **F.** Cell viability was determined after 48 h in multiple cell lines exposed to 500 nM OTX015 in combination with ibrutinib (2, 5, 10 μM), according to the ATPlite assay.

Since CEP28122 is a highly effective ALKi (EC50 of 20-30 nM) [29], we tested the effect of its combination with OTX015 (500 nM). Suboptimal concentrations (25-50 nM) were evaluated with a view to clinical trials designed to decrease ALKi pharmacological toxicities in the event that activity is seen with a low dose. CEP28122 at 25 nM in combination with OTX015 led to marked down-regulation of MYC protein, from very early time points (6 h, Figure 5A). Longer drug exposures were associated with a marked G1 cell cycle arrest although with minor cell growth inhibition.

We then evaluated the effect of selective inhibition of the SHH/GLI1 pathway, known to control cell growth in ALCL cells [7]. Exposure to OTX015 led to dose-dependent robust down-regulation of GLI1 following an initial spike in expression (Figure 5B). ALCL cells treated with combined OTX015 (500 nM) and GANT61 (5 μM), a selective GLI1/2 inhibitor [30], had higher rates of apoptosis compared to cells treated with either single agent drug (Figure 5C).

Having demonstrated that OTX015 led to interleukin-2-inducible kinase (ITK) down-regulation and given that deregulated ITK signaling is pathogenetic in some T-cell lymphomas [31, 32], we tested the therapeutic efficacy of ibrutinib, a selective inhibitor of Bruton's tyrosine kinase/ITK as a single agent or in combination with OTX015. Further support for this combination comes from preclinical studies showing strong synergy between BET inhibitors and ibrutinib in B-cell lymphoma [33]. As shown in Figure 5D, the dual drug combination led to substantial downregulation of pITK, and ibrutinib/OTX015-treated cells underwent growth inhibition followed by cell death (Figures 5D, 5E) [22, 34].

### OTX015 overcomes an ALK-refractory phenotype

**Figure 6:**
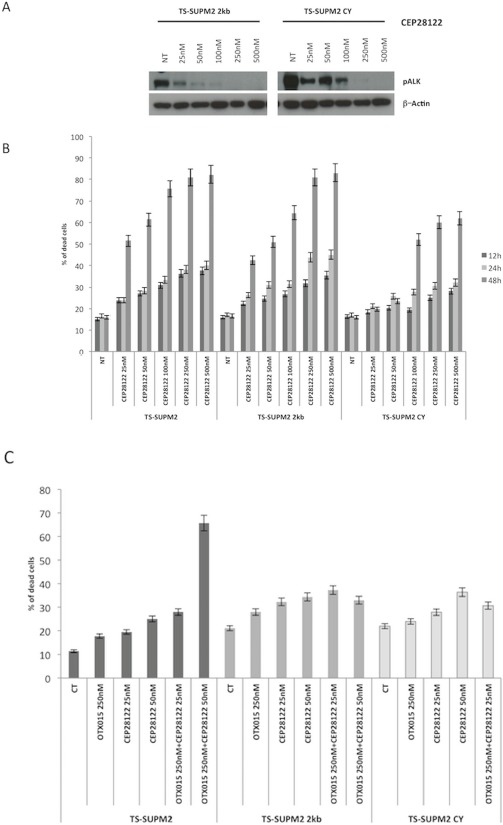

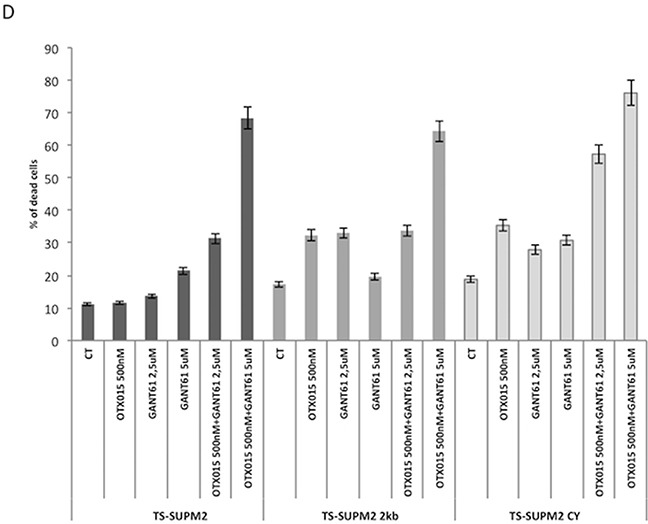
OTX015 overcomes ALK-refractory phenotype **A.** Phosphorylation levels of NPM-ALK in control (wild type NPM-ALK) and C1156Y NPM-ALK mutated transfected TS-Supm2 cells treated with a range of doses of CEP28122, according to Western blotting. β-actin was used as a loading control. NT, not treated control cells **B.** Percent cell death in cells (TS-Supm2, NPM-ALK wild type transfected, C1156Y-mutated) treated with CEP28122 at a range of concentrations for increasing exposure times (12, 24, 48 h) according to qRT-PCR. **C.** Combination treatment with OTX015 (250 nM) and CEP28122 (50 nM) in cells overexpressing wild type or NPM-ALK C1156Y mutation did not rescue drug resistance according to qRT-PCR. **D.** GANT61 and OTX015 combination treatment overcomes the resistance associated with the overexpression of wild type NPM-ALK or the C1156Y NPM-ALK mutated form.

One of the major obstacles in the chronic treatment of tyrosine kinase-driven cancers is the occurrence of drug-related refractory phenotypes, frequently found in populations bearing somatic mutations in or near the ATP-binding site of the kinase [9]. This phenomenon is observed with first and even second generation tyrosine kinase inhibitors. Drugs simultaneously blocking different pathways may thus represent a strategy to overcome tyrosine kinase inhibitor resistant phenotypes. Having demonstrated that OTX015 significantly increases the rate of apoptosis mediated by GANT61, we evaluated whether this combination could also effectively overcome the resistance mediated by a construct expressing an ALK-resistant NPM-ALK chimera in TS-Supm2 cells, mutated in position C1156Y. As expected, when these ALK-mutated cells were incubated with the ALKi CEP28122, they were more resistant compared to control transduced cells overexpressing wild type NPM-ALK, displaying higher levels of pALK in the presence of ALKi (Figure 6A). Interestingly, the combination of OTX015 with a low dose of CEP28122 was unable to overcome the resistant phenotype via the overexpression of either the wild type or mutated ALK (Figure 6B), however the combination of OTX15 and anti-ALK was effective (Figure 6C). The percentage of non-viable cells in TS-Supm2 cells overexpressing wild type or C1156Y NPM-ALK exposed to OTX015 (500 nM; 72 h) and anti-ALK or GANT61 alone (2.5 μM; 72 h) was analogous (Figure 6D). Conversely, the combination of OTX015 (500 nM) with a relatively low dose of GANT61 (2.5 μM; 72 h) was efficiently cytotoxic for TS-Supm2 cells overexpressing either C1156Y or wild type NPM-ALK (Figure 6D). These data suggest that this latter dual combination could represent a feasible alternative in the treatment of refractory ALK+ via gene amplification or ALK mutations.

## DISCUSSION

Our evaluations of the efficacy of OTX015, a new BRD inhibitor in early clinical development, in a panel of ALK+ ALCL cell lines revealed two key points; firstly that OTX015 had mainly cytostatic activity leading to G1 cell cycle arrest of ALK+ ALCL cells and inducing down-regulation of MYC and other transcription factors such as E2F1, as well as their target genes, and secondly that ibrutinib and GANT61 are synergistic with OTX015 leading to cell growth inhibition and cell death of ALKi resistant ALCL cells.

The development of specific BET inhibitors has recently led to intensive research activity exploring the molecular mechanisms of action and the clinical feasibility of this new class of compounds. To this end, the therapeutic utility of BET inhibitors has been proven in several hematopoietic diseases, including B-cell lymphoma [22, 34, 35] and acute lymphoblastic leukemia [36–39], however to our knowledge their value in PTCL has not been investigated. BET inhibitors have also emerged as powerful alternatives with therapeutic application in a variety of clinical arenas [38], including in the control of T-cell responses in the setting of graft-versus-host-disease [40] or in autoimmune conditions [41].

ALCL represents a well-defined group of PTCL for which several reliable cell line models are available, proving highly informative and playing a critical role in uncovering the molecular mechanisms leading to transformation [32]. We and others have taken advantage of these *in vitro* models to investigate pathways known to drive the neoplastic phenotype of primary ALCL [5]. To this end, it has been demonstrated that the JAK/STAT3 pathways play a critical role in the maintenance of the neoplastic phenotype of both ALK- [2] and ALK+ ALCL [42], and that its genomic ablation leads to cell death. It is associated with robust transcriptomic changes [27], with notably the loss of STAT3 signaling also leading to rapid down-regulation of MYC mRNA and protein levels [27]. The findings observed in our OTX015-treated ALCL cells are in line with our previous data and corroborate the hypothesis that MYC is a critical player of ALK signaling, sustaining cell cycle progression. As reported with other BRDi, MYC and its downstream genes are rapidly and reproducibly modulated by OTX015 treatment [16, 18, 22, 36]. However like others [16], we found that the phenotype induced by OTX015 cannot be overcome by MYC overexpression, suggesting that the efficacy of BET inhibitors is not exclusively due to MYC down-regulation. Indeed the forced expression of MYC overexpression in ALCL cells only delayed the therapeutic effects induced by OTX015. Further support for the validity of these data comes from the fact that they are in agreement with studies in AML cells treated with JQ1 [43]. Nonetheless, MYC expression levels represent a viable biomarker to assess the therapeutic efficacy of BET inhibitors.

Analysis of the transcriptome of ALK+ ALCL OTX015-treated cells provided insights into the mechanistic effect of OTX015. GEP showed that OTX015 affected a large pool of genes, which are also modulated by the treatment of other BET and HDAC inhibitors. Accordingly, OTX015 mediated the down-regulation not only of *MYC* and *E2F1* transcripts and their target genes, but also the up-regulation of various transcripts encoding several histones and a set of genes previously shown to be controlled by ALK/STAT3 signaling. Moreover we discovered that OTX015 could down-regulate expression of *ITK*, a kinase known to be overexpressed or deregulated via chimeric fusions in some T-cell neoplasms (36). ITK activation via TCR-mediated signaling leads to the activation of PCLγ and the nuclear translocation of nuclear factor of activated T cells [44]. Balanced activation of ITK has recently been demonstrated to be critical for T cell differentiation and appropriate development of Treg and T cells [32]. A cooperative effect of OTX015 and ibrutinib in ALK+ ALCL provides the rationale to explore BET inhibitors in T cell lymphoma as well as in auto-immune disorders. This hypothesis is supported by recent data describing the immunomodulatory effects of ibrutinib via ITK-inhibition in T cells [45]. Our discovery that the combination of ibrutinib and OTX015 increased cell death strongly supports the integration of this combination into the design of preclinical and clinical trials, particularly in the setting of chemo-refractory patients.

BET inhibitors often display cytostatic rather than cytotoxic activity. It is possible that the OTX015-induced up-regulation of oncogenes and pro-survival factors, such as JUNB or FOS (as shown by our GSEA analyses), might reduce the cytotoxic effect of this BET inhibitor. When we combined OTX015 with a large panel of drugs known to target either critical pathways in ALCL or that are broadly active in lymphoma, we identified several highly active combinations. This provides a window of opportunity to test new schedules and dosages with lower toxicity, or novel combinations that could potentially eradicate naïve tumors or overcome resistant phenotypes.

The recommended first-line therapy for ALK+ ALCL patients involves standard chemotherapy with doxorubicin-containing polychemotherapy [12]. Refractory or relapsed patients are now offered alternative treatments, including brentuximab vedotin either alone or in combination [46]. Solid responses are seen in refractory patients treated with ALKi, with prolonged clinical remission and ultimately allowing bone marrow transplantation [8]. Notably, the combination of OTX015 with crizotinib, one of several ALKi FDA-approved agents for the treatment of ALK+ non-small cell lung cancer, or with CEP28122, a selective ALKi [29], were found to be highly effective in our ALK+ ALCL cell lines. Thus, the combination of BET inhibitors and ALKi may have a relevant impact not only in the treatment of naïve ALCL but importantly also in patients with ALK+ solid tumors (who frequently display resistant mutated phenotypes [9]) or with a neoplasm counteracting ALK inhibition via alternative bypass mechanisms [47].

After prolonged treatment with ALKi, acquired resistance can emerge as a result of the selection and expansion of clones bearing activating mutations within the tyrosine kinase domain. These resistant phenotypes can be overcome by either second or third generation ALKi, or by compounds targeting alternative pathways and/or ALK downstream effectors. In light of this, we have shown that OTX015 down-regulates GLI1, a key mediator of the SHH/GLI1 pathway, and a protein known to be oncogenic in ALK+ ALCL [7]. We have also proven that the combination of GANT61 with OTX015 leads to a high rate of cell death, an outcome which is not seen with either individual drug. This novel combination thus represents a viable strategy for the treatment of resistant ALK+ neoplasms.

Collectively, our data demonstrate that OTX015 is an alternative and promising drug for the treatment of ALK+ ALCL, as a single agent or in combination with conventional therapy (CHOP) and/or selective compounds. Our data strengthen the potential role of BET inhibitors in this pathology, and encourage further drug discovery programs to identify new and effective BET inhibitors to associate with compounds currently in use for the treatment of lymphoma and/or with novel combinations. BET inhibitors are in their clinical infancy and highly effective and clinically manageable molecules are yet to be confirmed. OTX015 has favorable pharmacokinetic properties and manageable therapeutic properties with encouraging clinical activity reported in both acute leukemia and lymphoma patients [35, 39, 48]. This, along with the data presented here, makes this molecule an attractive clinical candidate for ALK+ ALCL and justifies the design of novel clinical trials employing OTX015 in association with inhibitors of ITK or SHH/GLI1 signaling for the treatment of relapsed or refractory ALK+ ALCL. It will be critical to identify those patients who may benefit the most along with biomarkers predicting or determining clinical responses in real-time. MYC appears to be a valuable marker for the selection of potential subsets, including up-front refractory ALK+ ALCL.

## MATERIALS AND METHODS

### Cell lines and compounds

ALK+ ALCL cell lines [4, 27, 28] and HEK 293T packaging cells (obtained from DSMZ, ATCC or were a gift from Dr Justus Duyster, University of Freiburg) were cultured under standard conditions (37°C in humidified atmosphere, with 5% CO_2_) in RPMI 1640 (Sigma-Aldrich) supplemented with 10% fetal calf serum (Lonza), 2 mM glutamine, 100 U/ml penicillin and 100 μg/ml streptomycin (Eurobio Biotechnology). We conducted monthly tests for mycoplasma and other contaminants. Cell line identification was confirmed by single-nucleotide polymorphism analysis (BioSynthesis).

OTX015 (MK-8628) was provided by Oncoethix SA (now Oncoethix GmbH, a wholly owned subsidiary of Merck Sharp & Dohme Corp., Lucerne, Switzerland). It was dissolved in DMSO as a stock solution of 10 mM and aliquots were stored at −80°C. Other drugs were obtained from the Weill Cornell Medical College pharmacy or from Selleckchem.

### MTT proliferation assay

Cells (2×10^4^ per well) were seeded in 96-well plates and grown for 24 h. OTX015 was serially diluted in tissue culture media, added to cells (in five replicates) at a range of concentrations (3 nM to 6 μM) and incubated for 72 h at 37°C. Control cells were treated with equivalent DMSO concentrations. 3-(4,5-dimethylthiazol-2-yl)-2,5-diphenyltetrazolium bromide (MTT, Sigma) was prepared as a 5 mg/ml stock in PBS and filter-sterilized. 0.5 mg/mL of MTT solution was added per well and cells were incubated in the dark at 37°C for 4 h then lysed with 25% SDS-based buffer. Absorbance was read at 570 nm on Beckman Coulter-AD340 (Beckman Coulter). At least three independent experiments were run for each cell line. Doses corresponding to the IC50 were estimated by fitting a sigmoidal model to the dose response curve using the R statistical package (www.r-project.org).

### Luminesence cell proliferation assay

Cells (2×10^4^ per well) were treated with OTX015 or equivalent DMSO concentrations then collected, diluted 1:1 with CellTiter-GLO Luminescent Cell Viability Assay reagent (Promega) and cell viability was measured using Perkin Elmer Luminometer, with the ATPlite assay. Results are expressed as RLU (relative luminescence units). Experiments were performed in triplicate and independently performed at least three times.

### Cell death and cell cycle analysis

Cells (1×10^5^/ml) were treated with a range of OTX015 doses (3 nM to 6 μM) and harvested at 24, 48 or 72 h. They were washed twice with cold PBS and stained with citrate buffer, propidium iodide (100 μg/ml) and RNase (10 mg/ml) for 15 min at room temperature. DNA content, cell cycle phase and rates of subG1 phase (dead cells) were determined using a FACScan or LSR II flow cytometer (Becton Dickinson). All flow cytometric analyses were performed using CellQuest Pro software (Becton Dickinson). Experiments were independently performed in triplicate at least three times.

### Senescence assay

Presence of senescent cells was evaluated with the Senescence β-Galactosidase Cell Staining Protocol kit (Cell Signaling Technology). Briefly, cells were harvested and washed with PBS, and fixed for 15 minutes at room temperature. After two PBS washes, β-Galactosidase Staining Solution was added and cells were incubated at 37°C overnight in a dry incubator. After 24 h, cells were washed, cyto-spun into a glass slide and evaluated under the microscope. β-galactosidase activity, seen as blue intracellular staining, was used as a senescence readout.

### Real-time polymerase chain reaction (RT-PCR)

RNA was extracted using Trizol (Invitrogen Life Technologies) reagent. The concentration of total RNA was determined using NanoDrop (NanoDrop Technologies). 500 ng of total RNA was treated with DNAseI recombinant, RNase-free (Roche Diagnostics) and reverse-transcribed using the Superscript First-Strand Synthesis System for RT-PCR kit (Invitrogen Life Technologies).

Quantitative RT-PCR (RT-qPCR) was performed with a Thermal iCycler (Bio-Rad) using the Bio-Rad iQ SYBR Green Supermix. PCR cycling conditions were 95°C for 5 minutes, followed by 40 cycles at 94°C for 10 seconds, and 60°C or 62°C for 30 seconds. Primer sets (Supplementary Table S1) were designed using Primer 3. To confirm the amplification specificity, the PCR products were subjected to the analysis of melting curve, linearity, and slope of standard curve. All PCR assays were performed in triplicate. Normalized gene expression results were calculated using as a reference glyceraldehyde-3-phosphate dehydrogenase (GAPDH) and/or human acidic ribosomal protein (HuPO) gene expression with the DCt method. Results are expressed as N-fold differences in target gene expression relative to GAPDH and/or HUPO.

### Western blotting analysis

Western blotting for protein expression analysis was performed as previously reported [2], using the following antibodies: anti-BRD2 (ab37633, AbCam), anti-BRD3 (ab56342, AbCam), anti-BRD4 (ab75898, AbCam), anti-MYC (9B11, Cell Signaling), anti-β-actin (Clone C4, Millipore) or anti-β-tubulin (Clone tub 2.1, Sigma), anti-pSHIP2 (#2007, Cell Signaling, Danvers, MA, USA), anti-pSTAT3 (#9145, Cell Signaling, Danvers, MA, USA), anti-pSRC (#1243, Cell Signaling, Danvers, MA, USA), anti-JunB (#3746, Cell Signaling, Danvers, MA, USA), anti-pITK (#3537, Cell Signaling, Danvers, MA, USA) and anti-Tubulin (#5666, Cell Signaling, Danvers, MA, USA).

### GEP and gene set enrichment analysis (GSEA)

GEP and GSEA data analysis were performed as previously reported [22].

### Drug combinations and evaluation of synergism

Cells (5×10^5^) were plated in triplicate in a 96-well plate and treated with a range of doses of several drugs (Supplementary Table S2) alone and in combination with OTX015 (500 nM). After 72 h drug exposure, 10 μl of CellTiterBlue (Promega) was added to each well. Plates were incubated at 37°C in humidified atmosphere with 5% CO2 for 1 h. Absorbance was read at 570 nm on a Beckman Coulter-AD340 (Beckman Coulter, Brea, CA, USA). IC50s were estimated by fitting a sigmoidal model through the dose-response curve using the Compusyn software. The combinations were evaluated using the Webb fractional product method [49], based on the equation Z = X + Y (1 − X) where Z is the expected effect of the combination and X and Y represent the effect of each drug alone. If Z is equal to the actual effect of the combination, then the relation is additive; if Z is higher, then it is less than additive, and if Z is lower, then it is more than additive (synergistic).

### Lentiviral infection for MYC overexpression

HEK 293T packaging cells were transfected with MYC [50] subcloned into the murine stem cell virus/green fluorescent protein (puro-MSCV-GFP) lentiviral vector (Addgene), in combination with third-generation helper plasmids [27]. The viral supernatant was collected after 48 h, filtered, and Karpas299 ALCL cells were transduced. After three cycles of infection cells were selected with puromycin (1 μg/ml, Sigma-Aldrich). Selected cells were treated with OTX015 or with an equivalent concentration of vehicle and harvested at different time points (24 to 72 h) after drug exposure. RNA and protein were extracted and processed for RT-PCR and Western blotting analysis, respectively. In addition, cell cycle analysis was performed in cells infected with MSCV-GFP-MYC or control non-transfected cells exposed to OTX015.

These are transduced cells with the control cassette.

### Lentiviral infection for ALKi resistance

Human NPM-ALK cDNA was generated by recombinant PCR amplification using specific primers and a high fidelity Taq polymerase. PCR products were first cloned into a pENTR1A no ccdB vector and subsequently transferred into the lentiviral vector pLenti PGK puro DEST using Gateway's LR reaction. ALKi resistance was conferred by introducing the C1156Y mutation. Mutagenesis was performed using QuickChange II Site-Directed Mutagenesis kit (Stratagene), according to the manufacturer's instructions. All constructs were DNA Sanger sequenced to verify their expected sequences.

HEK 293T packaging cells were transfected with NPM-ALK C1156Y (mutated) or NPM-ALK control (wildtype) lentiviral vector in combination with third-generation helper plasmids. The viral supernatant was collected after 48 h, filtered, and TS-Supm2 ALCL cells were infected. After three cycle of infection, infected cells were selected with puromycin (1 μg/ml). Selected cells were treated with CEP28122, OTX015 or GANT61 (single agent or in combination) or with an equivalent concentration of vehicle and harvested at different time points (12 to 72 h) after drug exposure. Protein was extracted for Western blotting or cells were harvested for cell cycle analysis.

## SUPPLEMENTARY FIGURES AND TABLES










