# Supplementary material for: Therapeutic efficacy of the bromodomain inhibitor OTX015/MK-8628 in ALK-positive anaplastic large cell lymphoma: an alternative modality to overcome resistant phenotypes
Source: Oncotarget. 2016 Oct 25;7(48):79637–53. doi: 10.18632/oncotarget.12876 (PMC5346742; doi:10.18632/oncotarget.12876)
Supplement: Supplementary file 1 [file oncotarget-07-79637-s001.pdf]

Therapeutic efficacy of the bromodomain inhibitor OTX015/MK-8628 in ALK-positive anaplastic large cell lymphoma: an alternative modality to overcome resistant phenotypes

SUPPLEMENTARY FIGURES AND TABLES

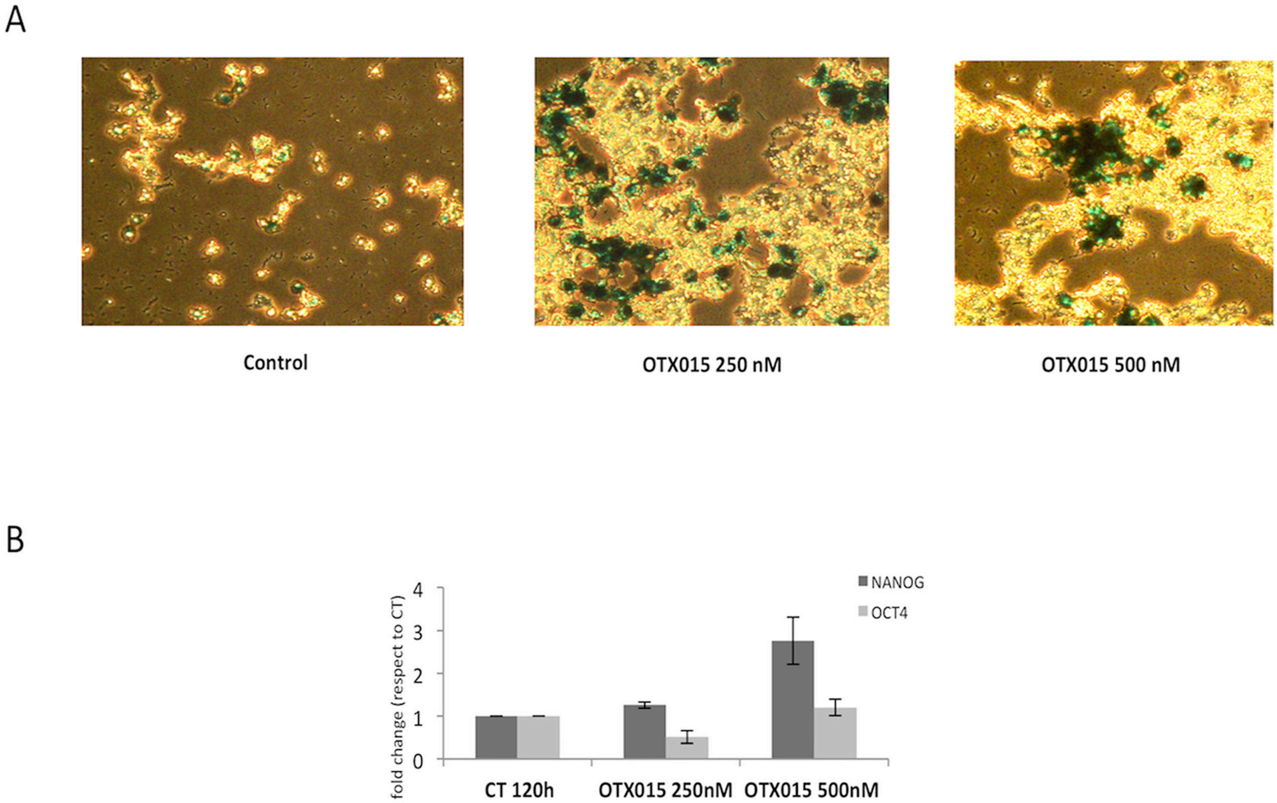

Supplementary Figure S1: OTX015 displays antiproliferative activity in ALK-positive ALCL *in vitro* models. **A.** Senescence was evaluated at 120 h post-OTX015 exposure using β-galactosidase staining. **B.** Relative expression of the transcription factors NANOG and OCT4 mRNA in OTX015-treated cells at 120 h post-treatment using RT-PCR. CT, DMSO-treated control.

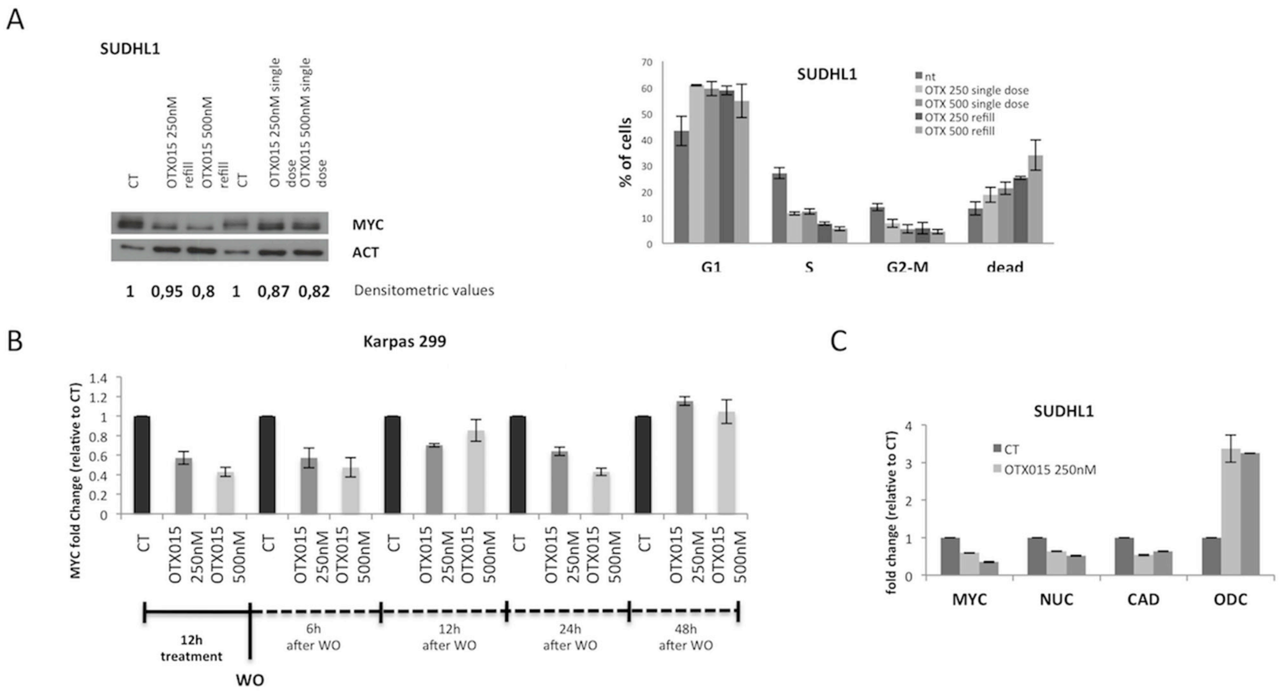

Supplementary Figure S2: OTX015 modulates MYC expression. A. Repeated OTX015 exposure (every 24 h) and single OTX015 exposure (72 h) gave comparable MYC protein down-regulation and increased accumulation of cells in G1 in cell cycle analysis. β-actin was used as a protein loading control. Densitometry values are indicated. **B.** OTX015 efficiently down-regulated MYC mRNA after a single short exposure. Cells were treated with OTX015 (12 h) and then cultured in complete media without OTX015 for a 'washout' period (WO) and harvested (6, 12, 24 and 48 h after washout). MYC mRNA expression was determined (RT-qPCR) and normalized to the GAPDH levels of corresponding samples. Fold-changes relative to control cells are reported. **C.** Changes in CAD, NUC and ODC mRNA expression evaluated by qRT-PCR after 24 h exposure to OTX015 (250 nM and 500 nM).

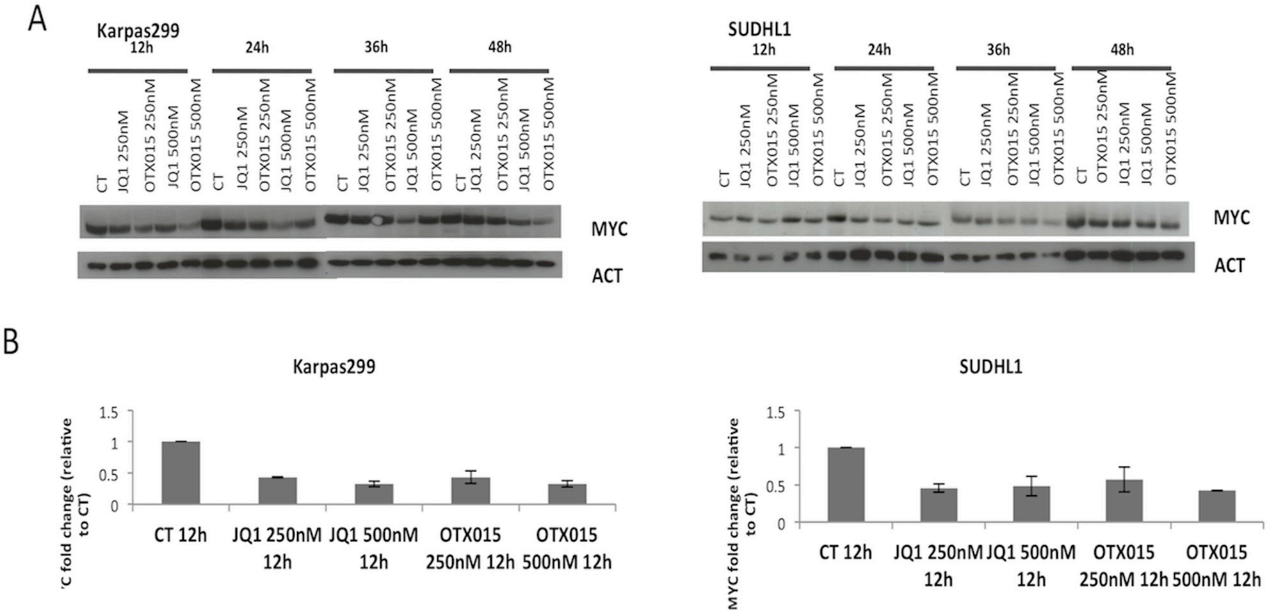

Supplementary Figure S3: OTX015 has comparable activity to the analog bromodomain inhibitor JQ1. A-B. OTX015 and JQ1 treatment (250 nM and 500 nM for 12, 24, 36 and 48 h) led to similar changes in MYC protein and mRNA expression evaluated by qRT-PCR and Western blot respectively in Karpas299 and SUDHL1 cell lines.

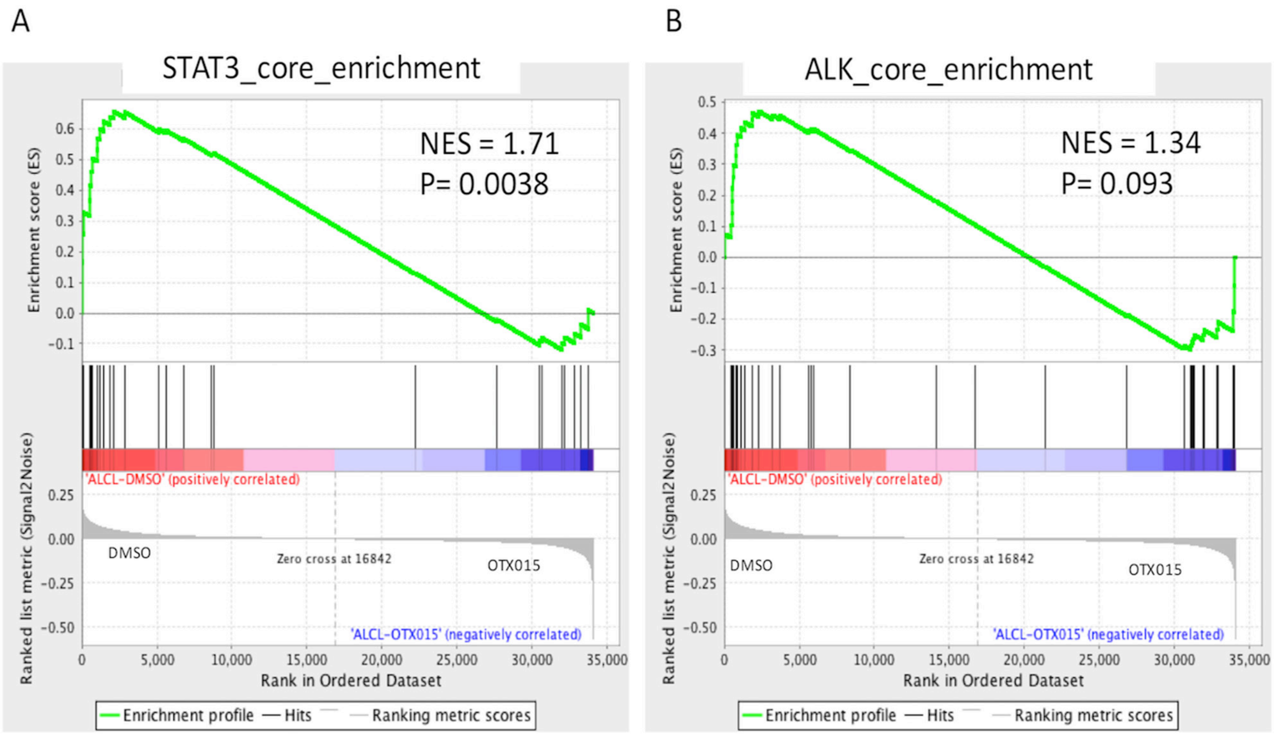

Supplementary Figure S4: Gene set enrichment analysis of cells treated with OTX015. Total RNA of the three cell lines was subjected to GEP followed by GSEA. Enrichment plots for gene sets obtained in **A.** from ALCL cells treated with shRNA STAT3 and **B.** from ALCL treated with ALKi or shRNA ALK. NES = normalized enrichment score. Piva R, Agnelli L, Pellegrino E, Todoerti K, Grosso V, Tamagno I, et al. Gene expression profiling uncovers molecular classifiers for the recognition of anaplastic large-cell lymphoma within peripheral T-cell neoplasms. J Clin Oncol. 2010;28:1583-90.

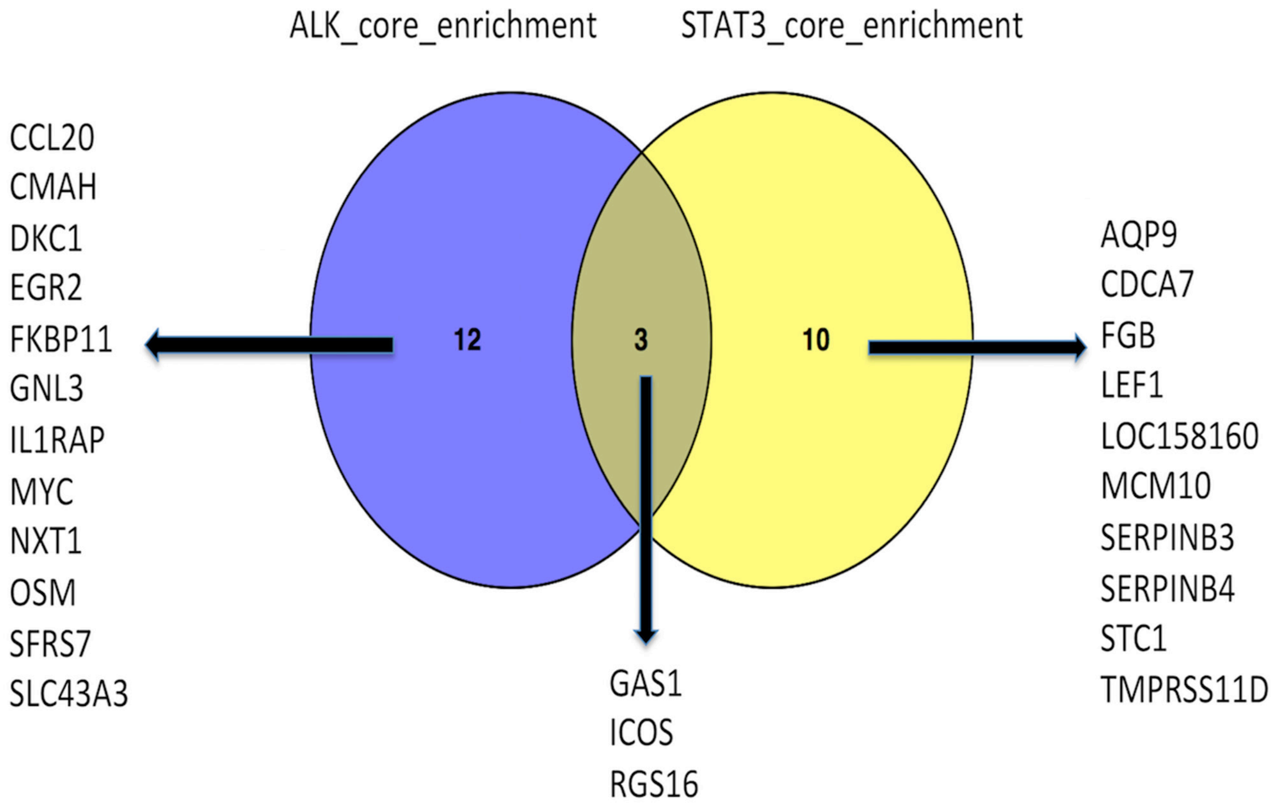

Supplementary Figure S5: OTX015 modulates a limited number of genes which are controlled via ALK-STAT3 signaling. Graphic representation of enriched genes after treatment of ALCL cells with 500 nM OTX015 known to be downstream of ALK or STAT3 signaling. Piva R, Agnelli L, Pellegrino E, Todoerti K, Grosso V, Tamagno I, et al. Gene expression profiling uncovers molecular classifiers for the recognition of anaplastic large-cell lymphoma within peripheral T-cell neoplasms. J Clin Oncol. 2010;28:1583-90.

Supplementary Table S1: Sequences of forward (F) and reverse (R) primers used for RT-PCR for BRD and signaling pathway genes

Gene	Primer sequence
BRD2-F	5'-ACTTGGCCTGCATGACTACC-3'
BRD2-R	5'-CTGTAGCTTTCGTGCCATTG-3'
BRD3-F	5'-CAACCATCACTGCAAACGTC-3'
BRD3-R	5'-GGGAGTGGTTGTGTCTGCTT-3'
BRD4-F	5'-AGTCATCCAGCACCACCATT-3'
BRD4-R	5'-TCTTAGGCTGGACGTTTTGC-3'
MYC-F	5'- GGACCCGCTTCTCTGAAAGG-3'
MYC-R	5'- TAACGTTGAGGGGCATCGTC -3'
NUC-F	5'-CAGAACCGACTACGGCTTTC-3'
NUC-R	5'-ACGCTTTCTCCAGGTCTTCA-3'
CAD-F	5'-CCCCAGATGAAATGGATGAG-3'
CAD-R	5'-CCCGAGTGTCTACTCCTTGC-3'
ODC-F	5'- GGTGCCTCCAGAGAGGATTA-3'
ODC-R	5'- TGGAATCATCAGTGGCAATC-3'
NANOG-F	5'- CAATGGTGTGACGCAGAAGG-3'
NANOG-R	5'-AAGGTTCCCAGTCGGGTTCA -3'
OCT4-F	5'- GCCCGAAAGAGAAAGCGAAC-3'
OCT4-R	5'- AACCACACTCGGACCACATC-3'
GLI1-F	5'- GAAGTCATACTCACGCCTCGAA-3'
GLI1-R	5'- AGCCAGGGAGCTTACATACAT-3'

Supplementary Table S2: Drugs used in the initial screening for synergism assays with OTX015

Drug	Target
(+)-JQ1	BET inhibitor
17-AAG	Hsp90 inhibitor
17-DMAG	Hsp90 inhibitor
2-DG	Glycolysis inhibitor
79-1085	BCL6 inhibitor
ABT-737	Bcl2 inhibitor
Crizotinib	ALK inhibitor
Bendamustine HCl	Alkylating agent
Bortezomib	Proteasome inhibitor
BS-181 HCl	CDK7 inhibitor
Carfilzomib	Proteasome inhibitor
VX-765	Caspases inhibitor
Cilengitide	RGD integrin inhibitor
CK37	Choline Kinase-α Inhibitor
Cytarabine	Antimetabolite agent
Doxorubicin	anthracycline antibiotics
Gemcitabine	Antimetabolite agent
GSK-126	EZH2 inhibitor
GSK-LSD1	LSD1 inhibitor
Imatinib	c-ABL inhibitor
Selinexor	XPO1 inhibitor
MI-2	MALT1 inhibitor
Obatoclax	Bcl2 inhibitor
Olaparib	PARP inhibitor
Panobinostat	HDAC inhibitor
Pralatrexate	Antimetabolite agent
PU-H71	Hsp90 inhibitor
Rapamycin	mTOR inhibitor
Ruxolitinib	JAK1/JAK2 inhibitor
Vorinostat	HDAC inhibitor
SNS-032	CDK7/9 inhibitor
Tiazofurin	IMP inhibitor
TIQ-A	PARP inhibitor
Tofacitinib	JAK3 inhibitor
TSA	HDAC class I inhibitor
Withaferin A	Multiple mechanisms
WS13	Multiple mechanisms
YK-5	Hsp70 inhibitor

Supplementary Table S3: GSEA summary data following 6 h exposure to 500 nM OTX015.

See Supplementary File 1

Supplementary Table S4: Ranked_gene_list_ALCL-DMSO_versus_ALCL-OTX015 following 6 h exposure to 500 nM OTX015.

See Supplementary File 2

Supplementary Table S5: Top 50 modified genes according to GSEA following 6 h exposure to 500 nM OTX015.

See Supplementary File 3

Supplementary Table S6: IC50 values were obtained with an MTT assay and Compusyn Analysis of four ALCL cell lines (TS-Supm2, SUDHL1, Karpas299 and DEL) treated with eight drugs as single agents (cilengitide, GSK-LSD1, tofacitinib, bendamustine, ruxolitinib, crizotinib, CEP28122 and GANT61; from 31.25 nM to 10 µM; first column per cell line), or in association with 500 nM OTX015 for 72 h (second column per cell line). For several agents, IC50 values were decreased after the combination treatment, suggesting an additive effect of the two drugs.

See Supplementary File 4
